# Genomic insights into the genetic diversity and genetic basis of body height in endangered Chinese Ningqiang ponies

**DOI:** 10.1186/s12864-025-11484-2

**Published:** 2025-03-24

**Authors:** Jiale Han, Hanrui Shao, Minhao Sun, Feng Gao, Qiaoyan Hu, Ge Yang, Halima Jafari, Na Li, Ruihua Dang

**Affiliations:** 1 Key Laboratory of Animal Genetics, Breeding and Reproduction of Shaanxi Province, College of Animal Science and Technology, Northwest A&F University, 712100 Yangling, China; 2 College of Information Engineering, Northwest A&F University, Yangling, 712100 China

**Keywords:** Ningqiang pony, Genetic diversity, Maternal origin, Genetic differentiation, Effective population size, Small stature

## Abstract

**Background:**

Genetic diversity in livestock and poultry is critical for adapting production systems to future challenges. However, inadequate management practices, particularly in developing countries, have led to the extinction or near extinction of several species. Understanding the genetic composition and historical background of local breeds is essential for their effective conservation and sustainable use. This study compared the genomes of 30 newly sequenced Ningqiang ponies with those of 56 other ponies and 104 horses to investigate genetic diversity, genetic differentiation, and the genetic basis of body height differences.

**Result:**

Population structure and genetic diversity analyses revealed that Ningqiang ponies belong to southwestern Chinese ponies. They exhibit a moderate level of inbreeding compared to other pony and horse breeds. Mitochondrial DNA analysis indicated that Ningqiang and Debao ponies share the dominant haplogroups A and C, suggesting a likely common maternal origin. Our study identified low genetic differentiation and detectable gene flow between Ningqiang ponies and Datong horses. The study also indicated the effective population size of Ningqiang ponies showed a downward trend. These findings potentially reflect the historical formation of Ningqiang ponies and population size changes. A selection signal scan (CLR and θπ) within Ningqiang ponies detected several key genes associated with bone development (*ANKRD11*, *OSGIN2*, *JUNB*, and *RPL13*) and immune response (*RIPK2*). The combination of genome-wide association analysis and selective signature analysis (*F*_ST_) revealed significant single nucleotide polymorphisms and selective genes associated with body height, with the most prominent finding being the *TBX3* gene on equine chromosome (ECA) 8. Additionally, *TBX5*, *ASAP1*, *CDK12*, *CA10*, and *CSMD1* were identified as important candidate genes for body height differences between ponies and horses.

**Conclusion:**

The results of this study elucidate the genetic diversity, genetic differentiation, and effective population size of Ningqiang ponies compared to other ponies and horses, further deepen the understanding of their small stature, and provide valuable insights into the conservation and breeding of local horse breeds in China.

**Supplementary Information:**

The online version contains supplementary material available at 10.1186/s12864-025-11484-2.

## Introduction

Horses (*Equus caballus*) were domesticated in the Central Asian steppes about 5,500 years ago [1, 2]. The high mobility of horses, combined with their widespread use by humans across extensive geographical areas since domestication, facilitated their broader distribution [3]. Horse breeds gradually diversified under the combined influences of natural environmental pressures and selective breeding. Domesticated horses are generally classified into three categories, including high-stature/heavy horses (draft breeds), light horses (riding breeds), and small-stature/low-weight horses (pony breeds)[4]. Variations in shoulder height and body type significantly affect the utility and fitness of these breeds. Pony breeds are typically defined as horses with a height of less than 14.2 hands (1.44 m) at the withers. They have small bone structures and are often used for children's horse riding [5].

Chinese ponies have attracted particular attention among global pony breeds due to their small stature. Typically, their shoulder height is less than 106 cm, which makes them well-suited for riding, recreation, leisure, and transportation in the mountainous regions of southwestern China [6]. Among these, Ningqiang pony stands out as one of China's five major pony breeds, alongside Jianchang pony, Guizhou pony, Debao pony, and Yunnan pony. The characteristics of Ningqiang pony are compact, tough, light and small, muscular and powerful, and this breed has played a significant role in human societies [7]. However, with the rapid advancement of agricultural mechanization and the transportation industry, the traditional roles of Ningqiang ponies have diminished. This shift has led to a drastic decline in their breeding numbers, pushing the breed at risk of endangerment [8]. Currently, limited research data presents a significant challenge to the development of scientific conservation programs for this breed.

The shoulder height and body type of a horse are important breeding objectives [9]. Over the years, the small stature of Ningqiang ponies has been investigated for various purposes, such as identifying screening markers for the body height of Ningqiang ponies through single nucleotide polymorphisms (SNPs)[10], comparing expression patterns of long non-coding RNA between ponies and horses to identify genes associated with the small stature of Ningqiang ponies [11], and revealing the intestinal flora associated with small stature of Ningqiang ponies [12]. A number of genes influencing body size in Chinese ponies and horses have been identified, such as *HMGA2*, *TBX3* [6], *LCORL*, *ZFAT*, *LASP1* [10], *CACNA1F* [13], and *NEL11* [4]. The advent of high-throughput sequencing technology has facilitated in-depth studies of the horse genome. Many studies have used whole-genome sequencing data to analyze the genetic diversity and adaptability of Chinese native horse breeds, offering critical insights into the scientific conservation efforts and breeding programs of these local breeds [14–16]. However, there are few reports on the whole-genome sequencing of Ningqiang ponies, and a more in-depth analysis of their genomic characteristics is lacking.

This study uses whole-genome sequencing data from 86 ponies and 104 horses, including 30 newly sequenced Ningqiang ponies, to assess their conservation status and effective population size, investigate the genetic basis of body height differences, and detect gene flow. The findings are expected to provide a comprehensive understanding of the genomics of Ningqiang ponies and contribute valuable knowledge for their breeding and conservation.

## Result

### Sequencing results and annotations

High-throughput sequencing generated genome data from 30 Ningqiang ponies with an average sequencing depth of about 17.4 × (Table S1). Additionally, whole-genome sequencing data from 7 previously published Ningqiang ponies were downloaded for comparative analysis. In this study, a total of 190 ponies and horses from 12 domestic breeds, including 37 Ningqiang ponies, were used for comparative analysis (Table S2). The average sequencing coverage across the data from 190 ponies and horses was approximately 11.64 × , with an average mapping rate of 98.77%. A total of approximately 23.76 million biallelic SNPs were annotated across 190 samples using ANNOVAR [17], with 16.08 million autosomal SNPs annotated in the sequenced Ningqiang ponies (Table S3).

### Population genetic structure

**Fig. 1:**
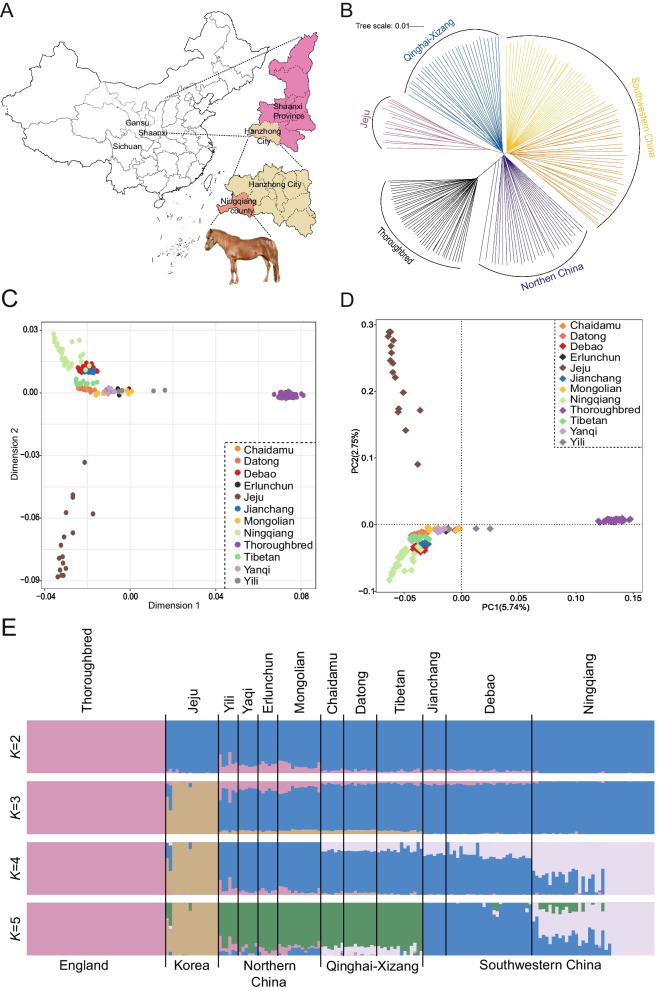
Population structure and relationship between Ningqiang pony and other pony and horse breeds. **A** Geographical distribution of Ningqiang pony. **B** Neighbor-joining tree of the relationships between Ningqiang pony and 11 other pony and horse breeds (The different breeds are clustered according to geographical location, and 37 Ningqiang ponies are marked yellow). **C** A Two-dimensional plot from multidimensional scaling analysis for 12 pony and horse breeds. **D** Principal component analysis of 12 pony and horse breeds (Different individuals are colored according to breeds). **E** Admixture analysis for ancestral genetic component sharing of Ningqiang pony and other breeds was included in the study (*K* = 2–5)

To gain a deeper understanding of the genetic background of Ningqiang pony, Neighbor-Joining (NJ) tree analysis, Multidimensional scaling (MDS) analysis, Principal Component Analysis (PCA), and ADMIXTURE analysis were employed to elucidate the genetic relationships between Ningqiang pony and 11 other pony and horse breeds. The NJ tree analysis indicated that Ningqiang ponies are part of the southwestern China clade (Fig. 1B). In the MDS plot of 190 ponies and horses (Fig. 1C), three distinct clusters were identified: Chinese pony and horse breeds clustered at the top left, Thoroughbred horses clustered at the top right, and Jeju ponies clustered at the bottom left. PCA showed three consistent clusters (Fig. 1D), with the first principal component (PC1) accounting for 5.74% of the total variation, primarily driven by differences between Thoroughbred horses and Asian ponies and horses. The second principal component (PC2) explained 2.75% of the total variation, distinguishing Jeju ponies from Chinese ponies and horses. Additionally, ADMIXTURE analysis on 12 breeds was conducted to assess the genetic affinity of Ningqiang ponies with other breeds (Fig. 1E). At *K* = 2, distinct ancestral origins were observed between Thoroughbred horses and Asian ponies and horses. At *K* = 3, Jeju ponies were differentiated from Chinese ponies and horses. At* K* = 4, a new ancestral origin of Ningqiang ponies appeared. At* K* = 5, ponies from southwestern China and Chinese horses showed separate ancestral origins. The cross-validation (CV) error was minimized at *K* = 3 (Fig. S1).

### Genetic diversity analysis

**Fig. 2:**
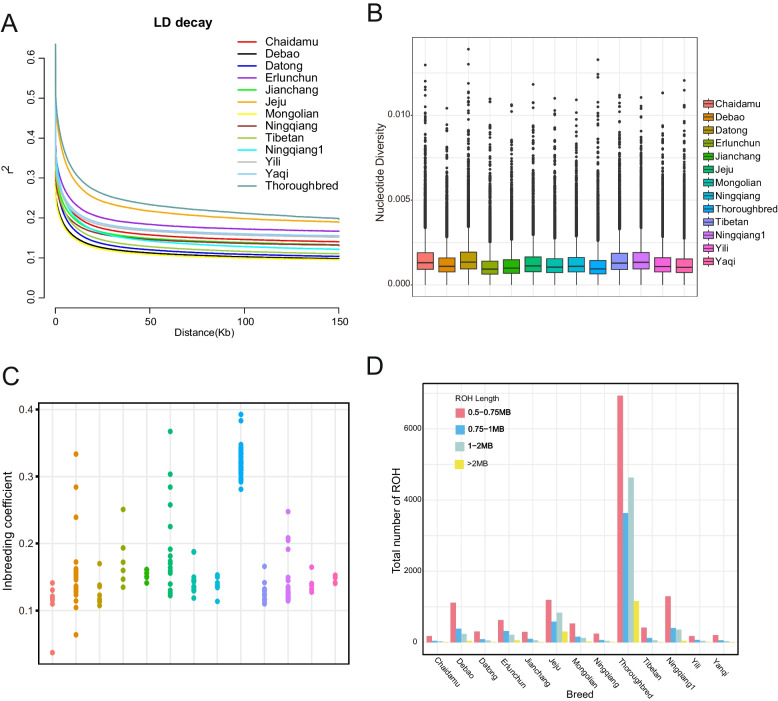
Autosomal genetic diversity of 12 breeds. **A** Linkage disequilibrium decay of horse autosomes estimated from 12 pony and horse breeds. "Ningqiang" refers to the downloaded Ningqiang ponies, while "Ningqiang1" refers to the sequenced Ningqiang ponies. **B** Nucleotide diversity of each breed. The horizontal line inside the box indicates the median of this distribution, and the points outside of the whiskers can be considered outliers. **C** Point plot of the inbreeding coefficient for each breed. **D** Estimation of the total number of runs of homozygosity (ROH) for each breed

The genetic diversity of 37 Ningqiang ponies and 11 other pony and horse breeds was compared and analyzed using multiple methods. To gain a deeper understanding of the breeding status of Ningqiang ponies in recent years, 37 ponies were divided into 30 sequenced Ningqiang ponies and 7 downloaded Ningqiang ponies. Subsequently, a similar number of individuals were randomly selected from each breed for linkage disequilibrium (LD) and nucleotide diversity (θπ) analysis (Table S4). LD analysis at the distance of 150 kb indicated that Thoroughbred horse exhibited the highest LD value, while Datong horse, Tibetan horse, sequenced Ningqiang pony, and downloaded Ningqiang pony had a relatively lower LD decay value (Fig. 2A). The θπ analysis showed that the nucleotide diversity of the sequenced Ningqiang pony (0.001504) was only lower than Datong horse (0.001521) and higher than that of other horse and pony breeds (Fig. 2B, Table S5). A total of 190 ponies and horses were used for the inbreeding coefficient (F_ROH_), observed and expected heterozygosity (Ho and He), and runs of homozygosity (ROH) analysis. The F_ROH_ reflects the degree of inbreeding within the population, indicating that Chaidamu horse had the lowest inbreeding (F_ROH_ = 0.110575). The inbreeding of the sequenced Ningqiang pony (F_ROH_ = 0.13761) was lower than the downloaded Ningqiang pony (F_ROH_ = 0.138135). In contrast, Thoroughbred horse had the highest inbreeding coefficient (F_ROH_ = 0.32172) (Fig. 2C, Table S6). The calculation of Ho and He showed that the Ho (0.1453) of the sequenced Ningqiang pony was slightly higher than the He (0.1412), while the Ho (0.1003) of the downloaded Ningqiang pony was significantly smaller than the He (0.1394)(Table S7). ROH analysis revealed that Jeju pony had the longest average ROH length, followed by Thoroughbred horse. The average ROH length of the sequenced Ningqiang pony was lower than that of Jianchang pony but higher than that of Debao pony and the downloaded Ningqiang pony. Chaidamu horse exhibited the shortest average ROH lengths (Fig. S2, Table S8). Among all the breeds studied, the length of the most abundant identified ROHs ranged from 0.5 to 0.75 Mb. Thoroughbred horse and Jeju pony exhibited longer and more numerous ROHs compared to Chinese pony and Chinese horse breeds. The sequenced Ningqiang pony had higher ROH lengths and numbers than the downloaded Ningqiang pony. In contrast, Chaidamu horse had the shortest and fewest ROHs (Fig. 2D).

### Comparative analysis of mitochondrial DNA in Ningqiang and Debao ponies

**Fig. 3:**
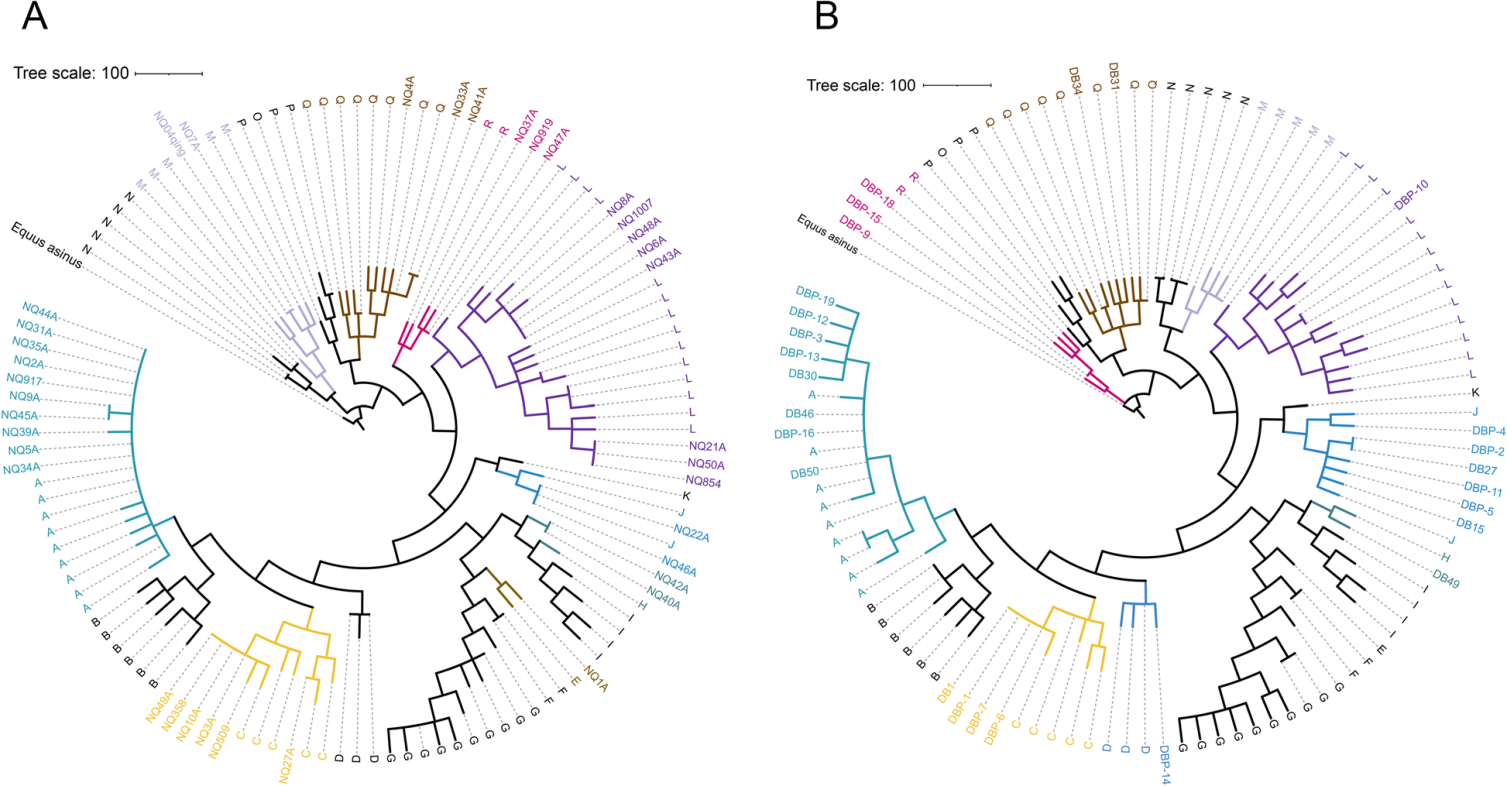
Mitochondrial genetic diversity of Ningqiang and Debao pony. **A** Phylogenetic tree of mitochondrial DNA from Ningqiang ponies, with individuals belonging to the same haplogroup represented by the same color. **B** Phylogenetic tree of mitochondrial DNA from Debao ponies, with individuals belonging to the same haplogroup represented by the same color

Mitochondrial DNA (mtDNA) is crucial for understanding the maternal origins of breeds. In this study, mtDNA data from 82 horses and one Asian wild donkey (*Equus asinus*) were collected for comparative analysis of the maternal origins of Ningqiang and Debao ponies (Table S9). The mtDNA sequences of the 82 horses represented 18 known haplogroups (A-R). The results revealed that the mtDNA of 37 Ningqiang ponies was assigned to haplogroups A, C, E, H, J, L, Q, M, and R, with haplogroups A (*n* = 10), L (*n* = 8), and C (*n* = 6) being the most prevalent (Fig. 3A). Similarly, the mtDNA of 26 Debao ponies was assigned to haplogroups A, C, D, H, J, L, Q, and R, with the most common haplogroups being A (*n* = 8), J (*n* = 6), and C (*n* = 4) (Fig. 3B).

### Genetic differentiation, genetic migration, and effective population sizes

**Fig. 4:**
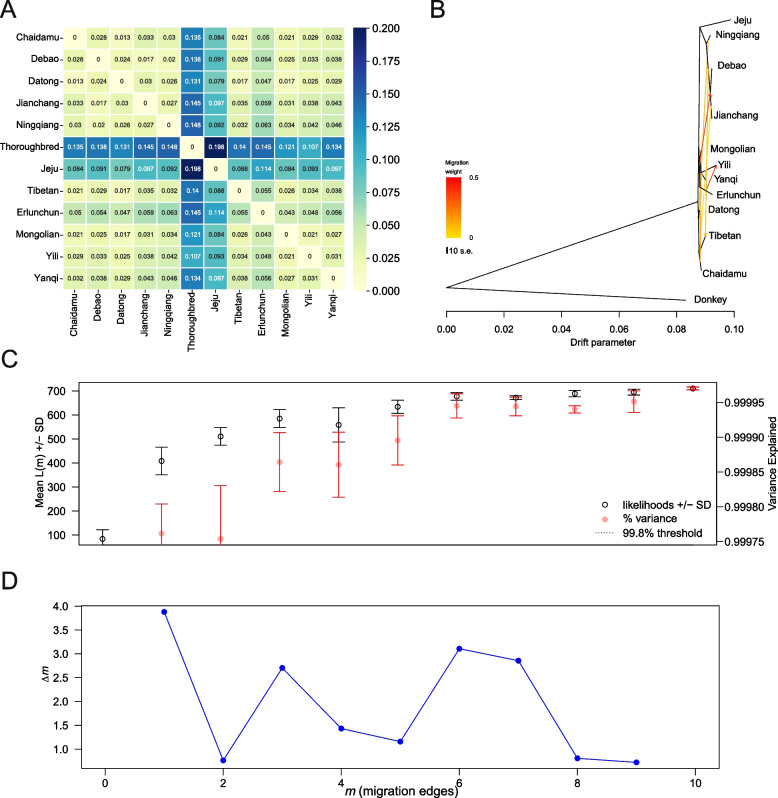
Genetic differentiation and gene flow among 12 pony and horse breeds. **A** Genetic distance of 12 pony and horse breeds. **B** Maximum Likelihood tree of Asian ponies and horses, with an Asian wild donkey as an outgroup, assuming six migration events (m = 6). **C** The output produced by OptM for the simulated dataset with m = 6 migration edges. The mean and standard deviation (SD) for the composite likelihood L(m) (left axis, black circles) and proportion of variance explained (right axis, red circles). The 99.8% threshold is not shown on the graph (dashed horizontal line). **D** The second-order rate of change (∆m) across values of m

The degree of genetic differentiation between Ningqiang pony and 11 other pony and horse breeds was calculated by fixation index (*F*_ST_), revealing that Ningqiang pony is genetically closest to Debao pony (*F*_ST_ = 0.02), followed by Datong horse (*F*_ST_ = 0.026) and Jianchang pony (*F*_ST_ = 0.027). In contrast, the furthest genetic differentiation was observed between Ningqiang pony and Thoroughbred horse (*F*_ST_ = 0.148)(Fig. 4A). To evaluate gene flow between Ningqiang pony and other Asian pony and horse breeds, a migration analysis was conducted using an Asian wild donkey as an outgroup (Table S2). The analysis showed that six migration events occurred under m = 6 (Fig. 4B, C, D). Relatively high admixture ratios were detected in the migration events from Mongolian horse to Jianchang pony and from Erlunchun horse to Yili horse. In contrast, the four other migration events from Chaidamu horse to Yanqi horse, Ningqiang pony to Datong horse, Jianchang pony to Tibetan horse, and Debao pony to Tibetan horse showed relatively low admixture ratios.

Moreover, the effective population size (*Ne*) of Ningqiang pony and four other pony and horse breeds were estimated (Fig. S3, Table S10). Surprisingly, from 101,215 to 9,814 years ago, the *Ne* of Ningqiang pony dropped dramatically, from approximately 1.208 million to about 4,270. Only from 5,805 to 5,476 years ago did the *Ne* show an increase. However, from around 3,000 years ago, the *Ne* of Ningqiang pony was significantly smaller than that of the other four pony and horse breeds.

### Selection signature in Ningqiang pony

**Fig. 5:**
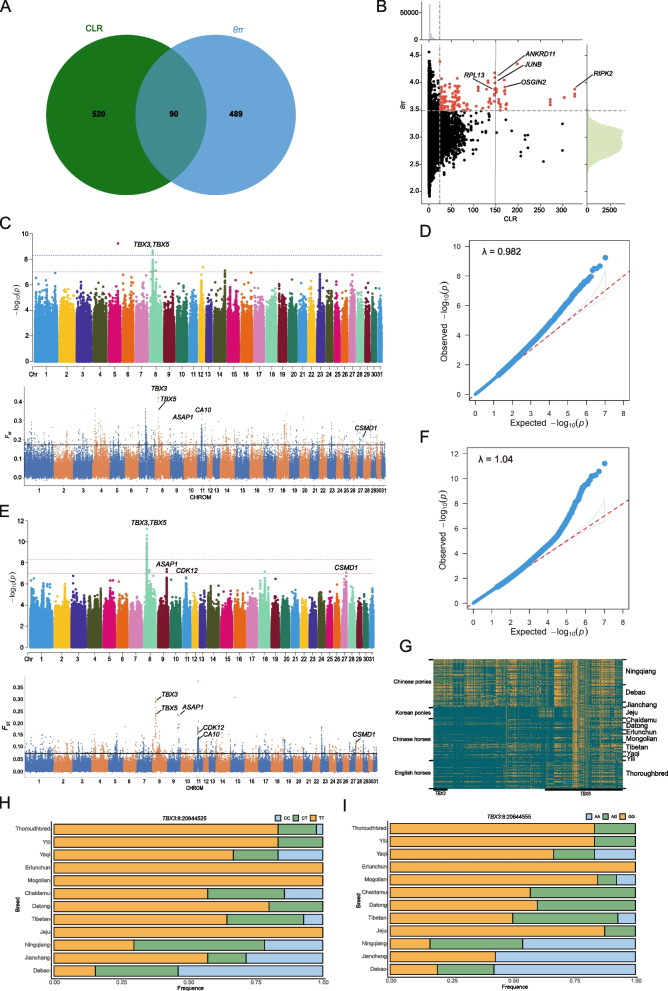
Genome-wide statistical analysis. **A** Venn diagram showing the overlap of genes identified by composite likelihood ratio (CLR) and nucleotide diversity (θπ) analyses. **B** Distribution maps of CLR and θπ values highlighting the selected signals within Ningqiang ponies. **C** Manhattan plot for the genome-wide association study of body height and analysis of signature selection in Ningqiang ponies (*N* = 37) and horses (*N* = 76). **D** QQ plot for the genome-wide association studies of Ningqiang ponies (*N* = 37) and horses (*N* = 76). **E** Manhattan plot for genome-wide association study of body height and analysis of signature selection in Chinese ponies (*N* = 74) and horses (*N* = 104). **F** QQ plot for the genome-wide association studies of 74 Chinese ponies and 104 horses. **G** The haplotype patterns of *TBX3* (ECA8: 20,639,011 bp-20,659,975 bp) and *TBX5* (ECA8: 20,804,678 bp-20,919,473 bp) gene. **H** Allele frequency of *TBX3* (ECA8:204,644,525) locus. CC homozygous is blue, CT heterozygous is green, and TT homozygous is orange. **I** Allele frequencies of *TBX3* (ECA8:204,644,555) loci. GG homozygous is blue, AG heterozygous is green, and AA homozygous is orange

The identification of selection signatures in Ningqiang ponies was performed using composite likelihood ratio (CLR) and θπ analyses. Both approaches revealed genes in the top 1% of windows as potential candidates for selection pressure. Specifically, CLR identified 610 genes, while θπ identified 579 genes, with 90 genes overlapping between the two analyses (Fig. 5A, B). This overlap suggests that these genes have likely undergone strong selective pressure in Ningqiang pony population. Among the candidate genes, *RIPK2* has been reported to be involved in innate and adaptive immunity in animals [18]. *ANKRD11* [19], *OSGIN2* [20], *JUNB* [21], and *RPL13* [22] have been previously reported to be associated with skeletal development in individuals, and may be part of the genetic basis for small stature in Ningqiang ponies.

### Genome-wide association studies and cross-population selection sweeps of Ningqiang ponies

To identify the loci associated with body height, genome-wide association studies (GWAS) were conducted using genetic data from 37 Ningqiang ponies and 76 typical horses (Table S11). The genome-wide significance threshold was calculated using Bonferroni correction [23], yielding a threshold of 4.78E-09 (*P* = 0.05/10,459,079), while the potential significance threshold was 9.56E-08 (*P* = 1/10,459,079). The False Discovery Rate (FDR) of significant SNPs was assessed using q-values less than 0.05 [24]. A total of 31 significant SNPs were identified with q-values less than 0.05, 5 of which met the genome-wide significance threshold (Fig. 5C, D, and Table S12). A significant genomic region was identified on ECA8 (ECA8:20.64 Mb-20.77 Mb), which includes the *TBX3* gene (ECA8:20,639,011 bp-20,659,975 bp). This region exhibits a strong degree of LD, indicating potential genetic significance (Fig. S4). Additionally, we noted that *TBX5* gene (ECA8:20,804,678 bp-20,919,473 bp) is located 36.94 kb away from a significant SNP (ECA8:20,767,738), and *F*_ST_ analysis between 37 Ningqiang ponies and 76 horses revealed that this gene exhibited a high selection signal value (Fig. 5C). Furthermore, the top 1% selection window identified by *F*_ST_ also annotated 428 other genes, including *TBX3*, *ASAP1*, *CA10*, and *CSMD1*. Notably, *TBX3* and *TBX5* have been previously reported as key candidate genes associated with body height differences in Chinese ponies and horses [6, 25]. Moreover, several additional genes were identified that may contribute to variations in body height among ponies and horses. For instance, *CA10* is implicated in bone metabolism and bone development [26], *ASAP1* is an important candidate gene affecting the growth and body size of Tibetan sheep [27], and biallelic variants of *CSMD1* cause developmental delay [28].

### Genome-wide association studies and cross-population selection sweeps of Chinese ponies

To validate the initial findings, population validation study was conducted on 74 Chinese ponies and 104 Chinese horses (Table S13). The whole-genome significance threshold was set at 4.81E-09 (*P* = 0.05/10,392,919) and the potential significance threshold was 9.62E-08 (*P* = 1/10,392,919). The GWAS identified 75 significant SNPs with q-values less than 0.05, of which 25 SNPs achieved genome-wide significance (Fig. 5E, F, Table S14). We identified an interesting region on ECA8 (ECA8:20.66 Mb-20.87 Mb) that contains the genes *TBX3* and *TBX5*. Considering the stringency of Bonferroni correction, we also examined other SNPs that did not reach the significance threshold but may still hold potential significance (Table S15). This approach revealed additional candidate genes, including *ASAP1* on ECA9 (ECA9:73,400,060 bp-73,747,813 bp), *CDK12* on ECA11 (ECA11:22,863,127 bp-22,961,193 bp), and *CSMD1* on ECA27(ECA27:35,739,655 bp-37,604,229 bp). Notably, *TBX3* and *TBX5* were strongly associated with body height traits and exhibited high selection signal values in *F*_ST_ analysis (Fig. 5E). Moreover, *TBX3* and *TBX5* exhibited different haplotypes in Chinese ponies compared to other ponies and horses, suggesting their potential as candidate genes influencing the body height difference between Chinese ponies and horses (Fig. 5G). In addition, *ASAP1*, *CDK12*, *CA10*, and *CSMD1* were also identified as significant candidate genes for body height differences between ponies and horses (Fig. 5E).

As previously reported, two SNPs (TBX3-EN1, ECA8:20,644,525 and TBX3-EN2, ECA8:20,644,555) are believed to play a significant role in regulating *TBX3* expression. In TBX3-EN1, the C allele is the ancestral type, while in TBX3-EN2, the A allele is the ancestral type [25]. The frequencies of these SNP alleles were examined in 190 ponies and horses. The ancestral alleles of these two SNPs were more frequent in Debao and Ningqiang ponies, whereas the mutant alleles were more common in Thoroughbred horses, Yili horses, Erlunchun horses, Mongolian horses, and Jeju ponies (Fig. 5H, I).

## Discussion

### The genetic relationship between Ningqiang ponies and other pony and horse breeds

China's horse breeding history spans over three millennia, with the current horse population exceeding 5 million, predominantly composed of indigenous breeds [29]. Among these, pony breeds represent unique local genetic resources, primarily distributed in the mountainous regions of southern and southwestern China [13]. Ningqiang pony is a breed classified within the southwestern China ponies, as supported by our NJ tree analysis. MDS plot and PCA results reveal that Chinese ponies and Chinese horses share a closer genetic relationship compared to foreign breeds, although there is still notable differentiation between the two. Genetic component analysis further indicates that the horse genome composition was influenced by geographical location. At *K* = 4, an independent ancestral component was identified in ponies from southwestern China. Although there is a certain probability of error, the possibility of horse domestication in southwestern China cannot be entirely ruled out [29, 30]. Additionally, northern China’s horses exhibited more significant genetic influence from foreign breeds, likely due to the introduction of foreign horse breeds into local populations to enhance specific phenotypic traits or performance characteristics [31].

### Analysis of autosomal genetic diversity

Different ecological conditions and human selection pressures have significantly influenced the morphology and genetic diversity of horses [32]. In recent decades, the demand for horses in transportation and agriculture has markedly diminished, leading to the near extinction of several breeds, including the Ningqiang pony. In response, the Chinese government established the Ningqiang National Conservation Farm to preserve this endangered breed [8]. Given the relatively small protected population and limited interaction with external populations, individuals within this group are theoretically more genetically homogeneous. However, the sequenced Ningqiang ponies exhibit high genetic diversity, which is higher than that observed in the downloaded Ningqiang ponies. This is evidenced by higher θπ values, smaller LD decay values, and a lower inbreeding coefficient, indicating that sequenced Ningqiang ponies possess greater genetic variation. This increased variation may enhance their resilience to external risks [33].

Additionally, we analyzed and compared the He and Ho of Ningqiang ponies with other pony and horse breeds. Notably, the sequenced Ningqiang ponies had the highest He and Ho values. Compared to downloaded Ningqiang ponies, the Ho of the sequenced Ningqiang ponies was significantly higher, suggesting a reduction in inbreeding and an increase in genetic diversity [34]. The close values of He and Ho among the sequenced ponies reflect that they face less selection pressure [35]. It is believed that the reduction of horse-power demand and mechanization has led to the decline of the genetic diversity of domestic horses, which has led to the increase of harmful alleles [36]. However, the sequenced Ningqiang ponies have a higher genetic diversity as the basis, showing that their genetic value cannot be ignored. Future conservation efforts should focus not only on preserving the excellent genetic traits of Ningqiang ponies but also on making reasonable use of these traits through activities such as riding.

### Analysis of mtDNA genetic diversity

Domestic horses possess the greatest number of maternal lineages among all domestic species, which is often taken as evidence that multiple domestications of horses have occurred [30]. Previous studies have demonstrated that horse mtDNA exhibits significant polymorphism and can be divided into 18 major haplogroups of A-R, with specific haplotypes associated with distinct geographical patterns. For instance, haplogroup A is most prevalent in Asia, gradually decreasing in frequency towards Europe, whereas haplogroup L is more common in European horses, with its frequency diminishing eastward [37]. In our study, both Ningqiang and Debao ponies were found to carry A and L haplogroups in their mtDNA data, and the locally dominant haplogroups may be related to the local domestication [29], this finding may suggest that these two breeds have a mixed origin of introduced horses and local wild horses [38]. Meanwhile, Ningqiang ponies have recently undergone a severe genetic bottleneck, which is likely to reduce their mtDNA genetic diversity to some extent [39]. A recent study has also documented a gradual reduction in the frequency of partial mtDNA haplogroups within local horse populations in China [30]. This decline may pose potential risks to the effectiveness of conservation strategies. Therefore, active measures should be taken to protect the genetic diversity of local horse breeds.

### Historical migration, genetic differentiation, and effective population size

Understanding genetic variation within and between breeds, as well as the evolutionary history of domesticated breeds, is crucial for providing conservation decision-making processes [40]. In this study, the* F*_ST_ value and Maximum Likelihood (ML) tree are used to reconstruct the genetic relationship between Ningqiang ponies and other Asian pony and horse breeds, which could reflect both historical connections and established genetic relationships [15]. Our results indicated genetic differentiation among Chinese pony breeds, and Debao Pony has the closest genetic distance to Ningqiang pony. At the same time, we noticed that Datong horse and Ningqiang pony were genetically close to each other. Additionally, gene flow was detected between Ningqiang pony and Datong horse from the Qinghai Province. Historical records suggest that Ningqiang pony may have originated from Qinghai horses that migrated south with the ancient Qiang people, and eventually evolved into the current breed of Ningqiang County [8]. The results of this study provide a reference for further research on the origin of Ningqiang pony, although its reference value is somewhat limited. Additional archaeological evidence is needed to fully understand the role of commercial trade and specific historical events in shaping the breed.

*Ne* is crucial for understanding the evolutionary history of a breed. A small *Ne* indicates limited genetic variation within the population, thereby increasing the likelihood of inbreeding [41]. Our results show that the *Ne* of Ningqiang pony has continuously declined over the past 101,215 years, reaching a low point around 5,805 years ago. Although there was a slight increase thereafter, Ningqiang pony still had the smallest *Ne* compared to the other four breeds around 1,000 years ago. This trend in *Ne* highlights the importance of conservation efforts for Ningqiang pony. However, given the potential for increased inbreeding due to low *Ne*, it is essential to implement breeding strategies that minimize the risk of inbreeding depression while preserving the unique genetic characteristics of this breed.

### Genome-wide selective signal scanning and GWAS

Shoulder height and body type have historically been a primary target of selection for breeders, and their significance as an economic trait in mammals has led to extensive research on its genetic underpinnings [42, 43]. Variants in the *HMGA*2 gene are widely believed to be linked to body height in western pony breeds, such as Shetland pony [44], Welsh pony [45], and Falabella pony [43]. However, it is notable that this mutation did not reach significant levels in southwestern China ponies, suggesting that the genetic basis of body size may differ between Chinese and Western pony breeds. In contrast, the *TBX3* gene has been identified as a key candidate associated with body height in Chinese horses [25]. *TBX3*, a T-box gene, plays a crucial role in limb development and is most significantly associated with the small stature of Debao pony [6]. Two SNPs in the enhancer region of the *TBX3* gene appear to be the principal contributors to height variation in Chinese horses, and they could explain up to 20.3% and 15.1% of shoulder height variations, respectively [25]. In our study, we calculated the frequencies of these two SNPs in 190 ponies and horses and found that the ancestral alleles of both SNPs were more frequent in Debao and Ningqiang ponies. Interestingly, the body height of Jeju pony appears to be influenced by different genetic mechanisms, as it exhibits a different haplotype with Chinese ponies in the *TBX3* gene, and this breed had a high mutation frequency of the two SNPs. In addition, the two SNPs have a high mutation frequency in Thoroughbred horses, suggesting that mutations in the *TBX3* enhancer may affect not only body height in Chinese horses but also in other horse breeds. Moreover, our study identified several other candidate genes, including *TBX5*, *ASAP1*, *CDK12*, *CA10*, and *CSMD1*. *TBX5* variants are thought to be associated with Holt-Oram syndrome, which exhibits variability in upper limb defects and congenital heart defects [46]. *ASAP1* is significantly associated with the growth of Tibetan sheep[27], and *ASAP1* loss leads to growth retardation and delayed ossification, as well as disorganization of cartilage and bone junction [47]. *CDK12* is a potential target for the treatment and prevention of osteoporosis, and overexpression of *CDK12* can inhibit osteoblast differentiation [48]. *CA10* plays a role in bone metabolism and is crucial for bone development in Zhaotong cattle [26]. *CSMD1 is* a multi-domain complement regulatory protein that shares homologs with many other proteins due to its Cub domain. These include several proteins involved in developmental regulation, such as bone morphogenetic proteins [49]. Since body height is largely determined by long bone growth and endocrine hormone signaling, the interaction between bones and endocrine organs is key in promoting growth [50, 51]. Therefore, these genes likely influence body height differences by regulating bone formation.

Despite these findings, the relatively limited sample size of this study may have reduced our statistical ability to identify SNPs with small to moderate effects. Moreover, the correction thresholds applied in this study may inadvertently exclude loci with weaker signals but still have biological significance. These limitations underscore the need for future research involving larger, more diverse populations of ponies and horses.

## Conclusion

Local breeds have valuable genomic traits accumulated through long-term natural and artificial selection, but these traits are increasingly influenced by commercial practices and mechanization. In this study, we comprehensively demonstrated the genomic characteristics and variations of Ningqiang ponies using whole-genome resequencing data, and examined their relationship with other pony and horse breeds. This provides a basis for the genetic evaluation of Ningqiang ponies. Additionally, we identified several important candidate genes associated with body height differences between Ningqiang ponies and horses, which have important implications for the breeding protection of small stature in Ningqiang ponies, as well as for animal breeding aimed at ideal height traits. Some of our findings provide an important foundation for the formulation of conservation strategies for Ningqiang pony, and also provide a reference for the scientific study of other endangered local breeds.

## Methods

### Sample collection and next-generation sequencing

We obtained blood samples from 30 Ningqiang ponies randomly collected at the Ningqiang National Conservation Farm in Ningqiang County, Shaanxi Province. Blood was collected from the jugular vein by a trained veterinarian, and no anesthesia or euthanasia was used during the procedure. The puncture site was disinfected with iodophor immediately after collection to prevent infection. Genomic DNA was extracted using the Rapid Blood Genomic DNA Extraction Kit (Tiangen Technology Co., Ltd., Beijing, China) and then randomly fragmented. A paired-end library with an average read length of 150 bp was constructed for each individual. Whole-genome sequencing was performed using the MGISEQ-2000 platform at the Wuhan BGI Sequencing Center.

We incorporated whole-genome data from multiple breeds to assess the genetic diversity of Ningqiang ponies and evaluated their genetic differentiation from other pony and horse breeds. Specifically, we collected whole-genome data from 7 Chinese horse breeds, including Chaidamu horses (*n* = 7), Datong horses (*n* = 10), Tibetan horses (*n* = 14), Erlunchun horses (*n* = 6), Mongolian horses (*n* = 13), Yili horses (*n* = 6), and Yanqi horses (*n* = 6). Genomic data from 3 Chinese pony breeds were also collected, including Debao ponies (*n* = 26), Jianchang ponies (*n* = 7), and Ningqiang ponies (*n* = 7). Furthermore, we used genomic data from 42 Thoroughbred horses and 16 Jeju ponies for comparison. This study used whole-genome data from 30 newly sequenced Ningqiang ponies, 56 previously sequenced ponies, and 104 horses from previous studies.

### Genome alignment, variant site acquisition, and annotation

The raw sequencing data were quality-controlled using Trimmomatic software [52] to obtain high-quality data. Subsequently, the clean reads from the 190 samples were mapped against the reference assembly EquCab 3.0 using the Burrows-Wheeler Aligner (BWA) with default parameters [53]. BAM files were sorted using the SortSam parameter and deduplicated using the MarkDuplicates parameter of Picard Tools (http://broadinstitute.github.io/picard). This was followed by variant calling with HaplotypeCaller from Genome Analysis Toolkit (GATK) v3.8 [54]. Then the data from 190 individuals, including data for nonvariant sites, were combined into one variant call format (VCF) file using GenotypeGVCFs from GATK. The GATK was also used for calling candidate SNPs, and SNPs were filtered using “VariantFiltration” with parameters set to Quality by Depth (QD) < 2.0, Fisher Strand (FS) > 60.0, Mapping Quality (MQ) < 40.0, MQRankSum < − 12.5, ReadPosRankSum < − 8.0, and Strand Odds Ratio (SOR) > 3.0, and the average sequencing depth of variants across all individuals set to ‘ < 1/3 × and > 3 × ’. Finally, the SNPs of each breed were annotated using ANNOVAR [17].

### Population structure and phylogenetic analysis

The NJ tree of 190 ponies and horses was constructed using PLINK v1.90 software [55] and MEGA v7.0 [56]. Prior to downstream analyses, SNPs in high pairwise LD were pruned using PLINK with the parameters (–indep-pairwise 1000 5 0.5), resulting in approximately 202.37 million SNPs retained. Genetic distances between individuals were calculated using PLINK, and the top 10 MDS dimensions were derived, the first two dimensions were then utilized to visualize the genetic distances. PCA was performed using the smartPCA of the EIGENSOFT [57], and population structure was inferred using ADMIXTURE [58] by testing *K* ranging from 2 to 5.

### Genetic diversity detection

The LD decay of the physical distance between SNPs was calculated and visualized using PopLDdecay software [59]. The θπ of each breed was estimated using VCFtools [60], with a window size of 50 kb and a step size of 50 kb. Additionally, VCFtools was also used to calculate the total length of ROH (L_ROH_) in the genome. The F_ROH_ value was estimated for each individual using the formula: F_ROH_ = L_ROH_/L_auto_, where L_auto_ is the total length of the autosomes in the horse genome (L_auto_ = 2,280,936,450) [33]. PLINK was used to calculate Ho and He for each breed. Genotype files were phased using Beagle 5 [61], and the phased data was used for ROH analysis (Table S16). The “–homozyg” option in PLINK was used to perform ROH analysis for each individual. The parameters were set as follows: (1) the sliding window size of 50 kb (–homozyg-window-snp 50); (2) the required minimum density was 1000 (–homozyg-density 1000); (3) allowance for one heterozygote per window (–homozyg-window-het 1); (4) allowance for up to one missing pairs per window (–homozyg-window-missing 1); (5) only ROH with a length greater than 500 kb is detected (–homozyg-kb 500). ROHs were divided into four categories based on length, including 0.5–0.75 Mb, 0.75–1 Mb, 1–2 Mb, and > 2 Mb.

### Genetic differentiation, migration detection, and effective population size

The smartPCA was utilized to calculate the* F*_ST_ values among 12 pony and horse breeds. TreeMix v1.12 [62] analyzed gene flow between Ningqiang pony and other Asian pony and horse breeds. Here, the number of migration events (-m) was set from 1 to 10, and three replicates were performed for each m value. An Asian wild ass was designated as the outgroup, and ML tree was constructed. The second-order rate of change of the m value (∆m) was used to determine the optimal number of migrations [63]. The historical effective population size of 5 breeds was simulated by SMC + + [64]. Each breed was randomly selected with 10 sample sizes (Table S10). The simulation was based on the previously reported mutation rate (mutation rate = 7.242e-9) and generation time (g = 8) [65].

### Genetic diversity and maternal origin of mtDNA in Ningqiang and Debao ponies

To investigate and compare the mtDNA genetic diversity of Ningqiang ponies and Debao ponies, the mtDNA genomes of 37 Ningqiang ponies and 26 Debao ponies were extracted from the whole-genome sequencing data using SAMtools [66]. The data in format was converted to FASTQ format using the SamToFastq module in Picard, and the FASTQ data for each individual was aligned to the horse mitochondrial reference genome (EquCab3.0) using Mapping-Iterative-Assembler [67], and the mtDNA genomes of 63 ponies were assembled. In addition, the mtDNA genomes of 82 domestic horses and one wild donkey, covering 18 known haplogroups, were downloaded and compared with the mitochondrial genomes of 63 ponies using MUSCLE v3.8.31 software [68]. Following this, the mtDNA genome sequences were analyzed using DnaSP v5.10 [69], and iTOL [70] was used for visualization purposes.

### Selective sweeps analysis and GWAS

We used CLR and θπ methods to test the selection signatures within the Ningqiang pony population. The CLR method based on SweepFinder2 [71], was applied to search for allele frequencies along the chromosome in a sliding window of 50 kb with a step size of 20 kb. VCFtools was used to calculate θπ by assessing the number of pairwise differences between sequences using the same window and step size as the CLR method. The top 1% of windows was considered a putative candidate signal of selection.

To identify gene loci associated with body height in Ningqiang ponies, we used PLINK software to filter SNPs from phased genotype file, SNPs with missing call rates > 10% across all individuals and minor allele frequencies (MAF) < 0.05 were filtered out, and 10,459,079 SNPs were obtained for subsequent analysis. A PCA based on the filtered SNPs was performed using PLINK software. Genome-wide Efficient Mixed Model Association (GEMMA) [72] was used to perform GWAS for body height in 37 Ningqiang ponies and 76 horses. The PC1 was used as a covariate, and the kinship matrix was used to correct for population kinship. The "CMplot" package in the R v4.4.2 environment was used to generate Manhattan and Quantile–Quantile (QQ) plots. The "qvalue" package in the R environment was used to calculate the FDR for significant SNPs [24]. To assess the presence of population stratification, the genomic inflation factor (λ) was calculated. This factor was estimated by converting the *P* values from the GWAS analysis to Z scores, squaring these Z scores, and then calculating the median of the squared Z scores. This median was divided by the theoretical median of the χ^2^ distribution with 1 degree of freedom (0.4549) [73]. The resulting ratio provides an indication of inflation in the GWAS. Additionally, the *F*_ST_ value between Ningqiang ponies and 76 horses was calculated using VCFtools, with a 50 kb sliding window and a step of 20 kb. The top 1% of windows were selected as the signal value.

To ensure the reliability of the results, GWAS and *F*_ST_ analyses were repeated on a larger cohort consisting of 74 Chinese ponies and 104 horses. The quality control conditions for the SNPs followed the same methodology described above, and a total of 10,392,919 SNPs were obtained. MLM implemented in GEMMA was used to perform GWAS in 74 Chinese ponies and 104 horses. The kinship matrix was used to correct for population kinship. No evidence of population stratification was found because the λ value was less than 1.05 (λ = 1.04). In addition, the whole-genome significance threshold was set at 4.82E-09 (*P* = 0.05/10,392,919) and the potential significance threshold was 9.62E-08 (*P* = 1/10,392,919).

## Supplementary Information

Supplementary Material 1.

Supplementary Material 2.

## Data Availability

The datasets supporting the conclusions of this article are available in the NCBI Database Sequence Read Archive, BioProject number PRJNA1127268.

## Abbreviations

ECA: Equine chromosome
SNPs: Single nucleotide polymorphisms
MDS: Multidimensional scaling
NJ: Neighbor-Joining
PCA: Principal Component Analysis
PC1: First principal component
PC2: Second principal component
ROH: Runs of homozygosity
LD: Linkage disequilibrium
θπ: Nucleotide diversity
Ho: Observed heterozygosity
He: Expected heterozygosity
F_ROH_: Inbreeding coefficient
L_ROH_: Length of ROH
mtDNA: Mitochondrial DNA
ML: Maximum Likelihood
*F*_ST_: Fixation index
CLR: Composite likelihood ratio
GWAS: Genome-wide association studies
GEMMA: Genome-wide Efficient Mixed Model Association
QQ plot: Quantile-Quantile plot
GATK: Genome Analysis Toolkit
BWA: Burrows-Wheeler Aligner
FDR: False Discovery Rate
VCF: Variant call format
CV: Cross-validation
Ne: Effective population size
∆m: The second-order rate of change of the m value
SD: Standard deviation
MAF: Minor allele frequencies
