# Supplementary material for: Genomic insights into the genetic diversity and genetic basis of body height in endangered Chinese Ningqiang ponies
Source: BMC Genomics. 2025 Mar 24;26:292. doi: 10.1186/s12864-025-11484-2 (PMC11934595; doi:10.1186/s12864-025-11484-2)
Supplement: Supplementary file 2 — Supplementary Material 2. [file 12864_2025_11484_MOESM2_ESM.docx]

**Genomic insights into the genetic diversity and genetic basis of body height in endangered Chinese Ningqiang ponies**

Jiale Han, Hanrui Shao, Minhao Sun, Feng Gao, Qiaoyan Hu, Ge Yang, Halima Jafari, Na Li, Ruihua Dang*

* Correspondence

Email: [dangruihua@nwsuaf.edu.cn](mailto:dangruihua@nwsuaf.edu.cn).


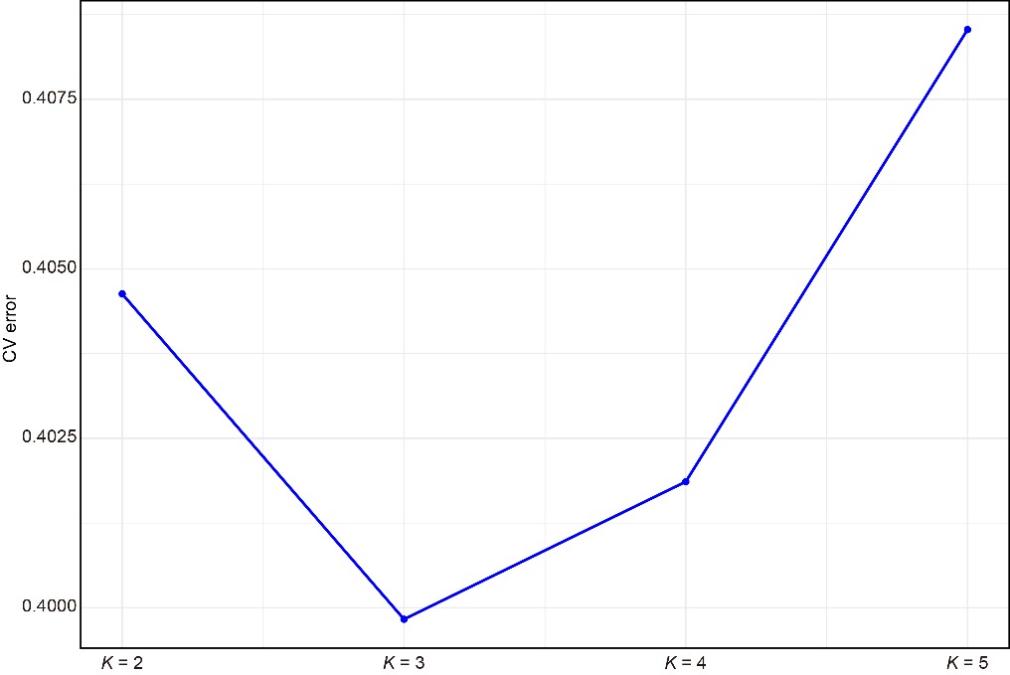


**Fig. S1** Line plots for different *K* and CV error values in ADMIXTURE analysis.


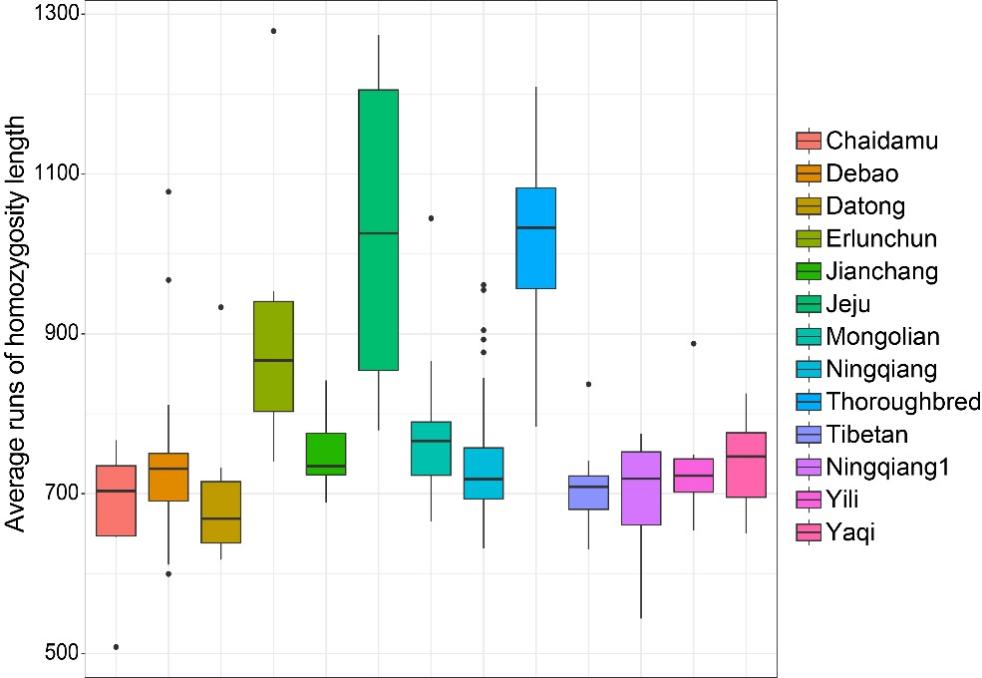


**Fig. S2** Box plot of average runs of homozygosity length. "Ningqiang" refers to the downloaded Ningqiang ponies, while "Ningqiang1" refers to the sequenced Ningqiang ponies.


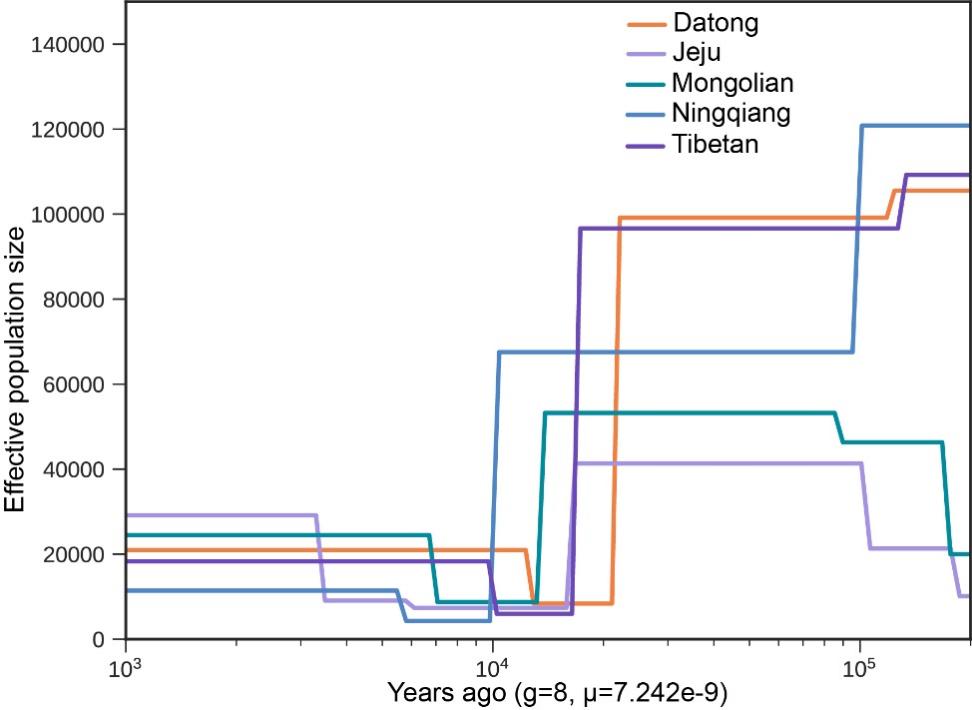


**Fig. S3** Effective population size of 10 Ningqiang ponies and 4 other pony and horse breeds.


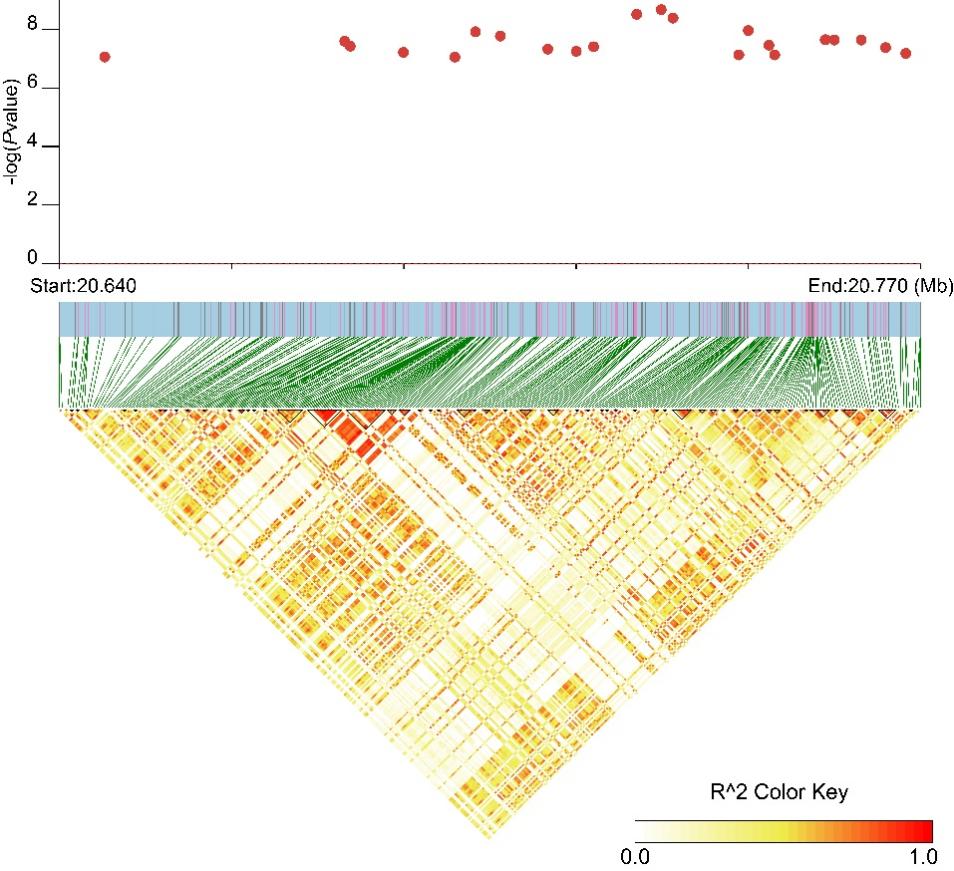


**Fig. S4** Strong linkage was observed around significant SNPs identified by GWAS in 37 Ningqiang ponies and 76 horses.
